# Concurrent Pseudomonas Periorbital Necrotizing Fasciitis and Endophthalmitis: A Case Report and Literature Review

**DOI:** 10.3390/pathogens10070854

**Published:** 2021-07-07

**Authors:** Yu-Kuei Lee, Chun-Chieh Lai

**Affiliations:** 1 Department of Ophthalmology, National Cheng Kung University Hospital, College of Medicine, National Cheng Kung University, Tainan 704, Taiwan; vincent11909@hotmail.com; 2 Institute of Clinical Medicine, College of Medicine, National Cheng Kung University, Tainan 704, Taiwan

**Keywords:** *Pseudomonas aeruginosa*, necrotizing fasciitis, endophthalmitis

## Abstract

(1) Background: Necrotizing fasciitis (NF) is an infection involving the superficial fascia and subcutaneous tissue. Endophthalmitis is an infection within the ocular ball. Herein we report a rare case of concurrent periorbital NF and endophthalmitis, caused by *Pseudomonas aeruginosa* (PA). We also conducted a literature review related to periorbital PA skin and soft-tissue infections. (2) Case presentation: A 62-year-old male had left upper eyelid swelling and redness; orbital cellulitis was diagnosed. During eyelid debridement, NF with the involvement of the upper Müller’s muscle and levator muscle was noted. The infection soon progressed to scleral ulcers and endophthalmitis. The eye developed phthisis bulbi, despite treatment with intravitreal antibiotics. (3) Conclusions: Immunocompromised individuals are more likely than immunocompetent hosts to be infected by PA. Although periorbital NF is uncommon due to the rich blood supply in the area, the possibility of PA infection should be considered in concurrent periorbital soft-tissue infection and endophthalmitis.

## 1. Introduction

Necrotizing fasciitis (NF) is a severe and rapidly progressive infection of the superficial fascia and subcutaneous tissue. Endophthalmitis is a type of severe ocular inflammation that occurs due to infection within the ocular ball. *Pseudomonas aeruginosa* (PA), a Gram-negative rod and opportunistic pathogen, is commonly found naturally in waste, water and soil [1]. Although PA is regarded as a common nosocomial pathogen, it also exists in the community environment and infects immunocompromised individuals [2,3]. PA infects the human body by attaching to soft tissue via its fimbriae and producing several surface-associated adherence factors or adhesins that promote attachment to epithelial cells and contribute to virulence [4]. PA then proliferates and releases enzymes such as elastase, alkaline protease and exotoxin A, which leads to tissue necrosis [5]. Therefore, PA may cause periorbital NF and other related complications, especially in immunocompromised patients, although previous reports showed that NF is generally caused by group A streptococci [6]. Accordingly, we report a case of concurrent periorbital NF and endophthalmitis caused by a community-acquired PA infection. As previous reports are relatively rare, we also conducted a literature review related to periorbital skin and soft-tissue infections caused by PA.

## 2. Results

**Figure 1:**
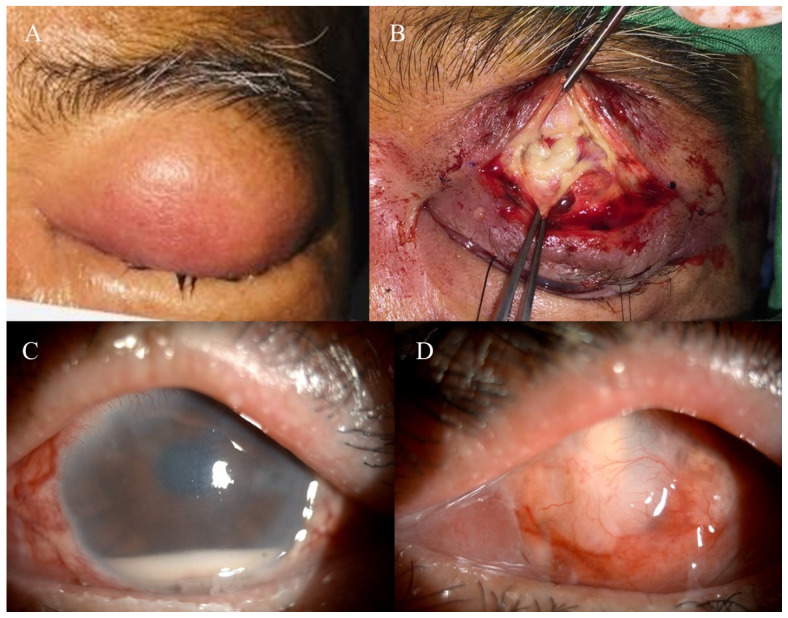
(**A**) The patient had periorbital pain and eyelid erythematous swelling for two days. (**B**) Whitish necrotic tissue in the upper palpebral conjunctiva, tarsus, Müller’s muscle and levator muscle was observed during surgery. (**C**) Slit lamp biomicroscopy revealed that the left eye injected the conjunctiva and hypopyon 3 mm in height. (**D**) After four months of follow-up, he developed phthisis bulbi.

A 62-year-old man presented with periorbital pain and erythematous eyelid swelling for two days (Figure 1A). The patient’s ocular history included bilateral vitreous hemorrhage and tractional retinal detachment status after pars plana vitrectomy four years prior. He had hypertension, hepatitis C and chronic kidney disease status three months after kidney transplantation. After kidney transplantation, he regularly took steroids and immunosuppressants. He had no other systemic conditions, such as diabetes mellitus, dyslipidemia, or a drug allergy.

The patient’s laboratory data revealed a white blood cell count of 1900/μL (normal range: 3400−9500/μL) with an absolute neutrophil count of 1041/μL (normal range: 1300−5600/μL), C-reactive protein level of 12.5 mg/L (normal range: <8.0 mg/L) and a fasting blood sugar level of 99 mg/dL (normal range: 60−99 mg/dL). The patient had regularly taken immunosuppressants after kidney transplantation, which may have led to leukopenia. The blood culture reports showed no bacterial growth. On ocular examination, the best-corrected visual acuity was 20/30 in the right eye and counting fingers at one meter in the left eye. The left upper eyelid was extremely swollen, firm and tender with erythematous changes. The conjunctiva was injected with chemosis. The anterior chamber, intraocular lens and fundus were unremarkable.

The patient was afebrile and then administered 400 mg intravenous teicoplanin every other day and 2000 mg ceftriaxone once a day. The steroids and immunosuppressants were gradually reduced. However, the eyelid swelling with tenderness persisted. Differential diagnoses, including preseptal cellulitis and orbital cellulitis, were considered. Initially, periorbital cellulitis was highly suspected based on the computed tomography images showing soft tissue swelling around the periorbital region. During the left eyelid incision and drainage, however, we noted large amounts of whitish necrotic tissue in the upper palpebral conjunctiva, tarsus, Müller’s muscle and levator muscle (Figure 1B). The pathology of these necrotic tissues showed suppurative inflammation composed of dense neutrophilic infiltration with focal necrosis. The definite diagnosis of periorbital NF was made based on the surgical and pathological findings. A microbial culture from the necrotic tissue was performed during the operation and revealed a PA infection. The intravenous antibiotics were switched to 2000 mg ceftazidime once a day. An anterior orbitotomy with debridement was performed again with removal of necrotic tissue. He was discharged five days after the surgery against the physician’s advice.

After the loss of follow-up for three weeks, he returned again due to left-eye tenderness. The left-eye best-corrected visual acuity decreased to only light perception. Left eyelid wound dehiscence with discharge was noted. The slit lamp biomicroscope revealed a left-eye hypopyon 3 mm in height (Figure 1C). The fundus was veiled, and ophthalmic sonography showed vitreous opacity. A left eyelid wound debridement with intravitreal vancomycin (1 mg/0.1 mL) and ceftazidime (2 mg/0.1 mL) was performed due to suspected left-eye periorbital NF and endophthalmitis. The reports of cultures of intraocular contents, including vitreous and aqueous humor, both showed a PA infection. After another four intravitreal ceftazidime injections according to the culture report, the tenderness improved. There were two scleral ulcers near the limbus, and the culture of discharge from these ulcers also showed growth of PA. After four months, the scleral ulcer in the left eye gradually improved. Visual acuity had reached the level of no light perception, with notable phthisis bulbi (Figure 1D).

## 3. Discussion

NF is a severe infection with a rapidly progressive infectious process involving the superficial fascia and subcutaneous tissue. Its clinical course is often rapidly progressive, with a high morbidity and mortality rate and the infection site is usually located in the extremities [7]. However, periorbital NF is uncommon due to the rich blood supply in the area [6]. The most common pathogens responsible for periorbital NF belong to monomicrobial group A streptococci [6], whereas causal microorganisms of NF elsewhere are often polymicrobial, with group A streptococci and Enterobacteriaceae being the most common pathogens [7]. In our case, instead of streptococci, the microbial cultures from the periorbital tissue, scleral ulcers, aqueous humor and vitreous all showed a PA infection. Moreover, the patient did not have signs of toxic shock syndrome (e.g., fever, skin rash and low blood pressure), which may cause the high mortality rate in patients with NF infected by group A streptococci [6]. Notably, the patient we reported is the first case of concurrent periorbital NF and endophthalmitis caused by PA.

Endophthalmitis is a type of severe ocular inflammation that occurs due to infections within the ocular ball and can be classified as exogenous or endogenous based on the transmission route of the infection [8]. Exogenous endophthalmitis occurs when the infecting organisms directly enter the eye, while endogenous endophthalmitis results from infectious agent hematogenous seeding of the eye. In the literature, septic metastatic endophthalmitis may develop from distant NF of a lower extremity infected by *Klebsiella pneumoniae* [9]. Severe infections, such as liver abscesses and bacteremia, can also cause endogenous endophthalmitis concurrent with orbital cellulitis or NF of the leg [10,11,12,13]. In contrast, based on the clinical manifestations and culture findings in our patient, we surmised that the endophthalmitis was exogenous, and was caused by direct invasion of the NF of the periorbital tissue through the ulcerative sclera. The leading causative organisms of endophthalmitis are Gram-positive bacteria, but endophthalmitis caused by PA is usually associated with poor visual prognosis, even if immediate intravitreal antibiotic treatment is prescribed [14]. Similarly, the delayed administration of antipseudomonal agents resulted in unfavorable prognoses in our case.

Herein, we reviewed 16 studies on the topic of periorbital skin and soft-tissue infections caused by PA (Table 1). Of these patients, only three with comorbid leukemia, lung cancer and status post renal transplant were diagnosed with NF. Another three were recognized as concurrent orbital cellulitis and endophthalmitis. Of these three patients, one had drug-induced neutropenia, and the other two had iatrogenic causes after radial keratotomy and cataract surgery. Of importance, the patient we indicated is an unusual case of concurrent periorbital PA NF and endophthalmitis and progression from periorbital cellulitis due to the delayed appropriate antimicrobial therapy. Furthermore, of these reviewed patients, most patients had a relatively immunocompromised status, including diabetes mellitus, Felty syndrome, lung cancer or leukemia with chemotherapy, drug-induced neutropenia, systemic lupus erythematosus and renal transplant with immunomodulatory medications. Our patient had the same immunosuppressed status after a kidney transplantation as the patient reported by Lim et al. [15].

Among the patients described, the necrotizing regions mostly involved the eyelid, conjunctiva and canthus. However, four patients experienced necrotization and the destruction of the lacrimal system. The treatments for PA-related periorbital soft-tissue infection comprised intravenous antibiotics and necrosis tissue debridement. Two patients who received early treatment died due to sepsis and small-cell lung carcinoma. For the patients with a controlled infection, most developed eyelid defects or lagophthalmos that needed further eyelid reconstruction surgery. Epiphora could be noted in patients with lacrimal system necrosis. For the patients with an infection involving the lacrimal drainage system, one of whom had undergone necrosis debridement and silicone tube intubation of the lacrimal duct. Early silicone tube intubation can prevent secondary lacrimal duct occlusion [5]. The patient had a patent lacrimal drainage system after a one-year follow-up. Another patient who developed bilateral lacrimal duct occlusion and persistent epiphora had undergone a bilateral conjunctivodacryocystorhinostomy [16].

Periorbital Pseudomonas NF is often missed early because symptoms are similar to those of other common soft-tissue infections [6]. As in our review (Table 1), major comorbidities were relatively immunocompromised. Especially for these immunocompromised individuals, NF should be considered when the patient does not respond to standard antimicrobial therapy. Due to the lack of culture reports in the early stage, medical management alone may result in delayed treatment with fatal consequences in cases diagnosed with skin and soft-tissue infections. For cases of suspected NF, the gold standard treatment remains early surgical exploration with tissue debridement as required and systemic broad-spectrum anti-PA antimicrobial therapy [17].

## 4. Conclusions

When periorbital skin and soft-tissue infections are concurrent with endophthalmitis, the possibility of PA infections should be considered. A delayed diagnosis and administration of the appropriate antibiotics for PA infections may cause poor prognosis and possible fatal consequences. When PA NF is suspected, urgent surgical debridement together with systemic antimicrobial therapy should be administered as early as possible. Accordingly, more research should be conducted to determine the risk factors for PA infections in patients experiencing periorbital skin and soft-tissue infections, particularly in NF, which can help us to prompt and appropriately treat this difficult disease.

## Figures and Tables

**Table 1:** Periorbital skin and soft tissue infections caused by *Pseudomonas aeruginosa*.

Reference **	Age */Sex	Eye	Involved Region	Diagnosis	Predisposing Factors or Comorbidities	Bacteremia	Sequelae
Steinkogler, 1988 [18]	6 weeks/M	OU	Eyelid, lacrimal system	Eyelid necrosis	Idiopathic	Positive	Medial canthal displacement, lagophthalmos, corneal perforation
McLeod, 1995 [19]	49/M	OD	Orbit, ocular globe	Orbital cellulitis, endophthalmitis	Radial keratotomy	N.A.	Phthisis bulbi
Lattman, 1998 [16]	62/F	OU	Eyelid, canthus, lacrimal system	Eyelid necrosis	Diabetes mellitus	Negative	Eyelid defect, epiphora
Comaish, 2000 [20]	82/F	OD	Eyelid, conjunctiva	Eyelid necrosis	Peripheral vascular disease	Positive	Eyelid defect
Dickenson, 2002 [21]	70/M	OU	Eyelid, conjunctiva	Eyelid necrosis	None	Positive	Eyelid defect, epiphora
Watson, 2003 [22]	70/F	OU	Eyelid, canthus, lacrimal system	Eyelid necrosis	Systemic lupus erythematosus, leucopenia	Negative	Eyelid defect, epiphora
Poitelea, 2005 [23]	68/M	OU	Eyelid, conjunctiva	Eyelid necrosis, necrotizing fasciitis	Chronic lymphocytic leukemia, insect bite	N.A.	Lagophthalmos
Ganesh, 2007 [24]	14 days/M	OS	Eyelid, canthus, lacrimal system	Eyelid necrosis	Congenital nasolacrimal duct obstruction, leukocyte adhesion deficiency type I	N.A.	Hard-palate perforation
Luemsamran, 2008 [12]	75/F	OS	Orbit, ocular globe	Orbital cellulitis, endophthalmitis	Drug-induced neutropenia	Positive	Light perception
West, 2008 [25]	68/M	OU	Eyelid, conjunctiva	Eyelid necrosis	Felty syndrome	Negative	Eyelid defect
Hulten, 2009 [1]	60/M	OS	Eyelid, conjunctiva	Eyelid necrosis	Large B-cell lymphoma, acquired immunodeficiency syndrome	Negative	Sepsis, death
Decock, 2010 [26]	74/M	OS	Orbit, ocular globe	Orbital cellulitis, endophthalmitis	Cataract surgery	N.A.	Enucleation
Lim, 2010 [15]	22/F	OS	Eyelid	Eyelid necrosis, necrotizing fasciitis	Congenital bilateral dysplastic kidneys status post renal transplant	Negative	Eyelid defect, necrotizing fasciitis with toxic shock
González, 2013 [17]	53/M	OU	Eyelid, conjunctiva, cornea	Eyelid necrosis, necrotizing fasciitis	Small cell lung carcinoma	Negative	Left eye enucleation, death
Kim, 2014 [27]	48/F	OU	Eyelid, canthus, lacrimal system	Eyelid necrosis	Systemic lupus erythematosus, acute promyelocytic leukemia	Negative	Eyelid defect
Yeh, 2019 [5]	14/F	OD	Eyelid, canthus, lacrimal system	Eyelid necrosis	Systemic lupus erythematosus	Negative	None

** Ordered by publication year; * years of age if no unit is given; Abbreviations: F: female, M: male, OD: right eye, OS: left eye, OU: bilateral eyes, N.A.: not mentioned in the article.

## Data Availability

The datasets used and/or analyzed in the course of the current study are available from the corresponding author on reasonable request.
